# Do Executive Attentional Processes Uniquely or Commonly Explain Psychometric *g* and Correlations in the Positive Manifold? A Structural Equation Modeling and Network-Analysis Approach to Investigate the Process Overlap Theory

**DOI:** 10.3390/jintelligence9030037

**Published:** 2021-07-15

**Authors:** Stefan J. Troche, Helene M. von Gugelberg, Olivier Pahud, Thomas H. Rammsayer

**Affiliations:** Institute of Psychology, University of Bern, Fabrikstrasse 8, CH-3012 Bern, Switzerland; helene.vongugelberg@psy.unibe.ch (H.M.v.G.); olivier.pahud@bfs.admin.ch (O.P.); thomas.rammsayer@psy.unibe.ch (T.H.R.)

**Keywords:** psychometric intelligence, positive manifold, *g* factor, executive attention, process overlap theory, mental speed

## Abstract

One of the best-established findings in intelligence research is the pattern of positive correlations among various intelligence tests. Although this so-called positive manifold became the conceptual foundation of many theoretical accounts of intelligence, the very nature of it has remained unclear. Only recently, *Process Overlap Theory* (POT) proposed that the positive manifold originated from overlapping domain-general, executive processes. To test this assumption, the functional relationship between different aspects of executive attention and the positive manifold was investigated by re-analyzing an existing dataset (N = 228). Psychometric reasoning, speed, and memory performance were assessed by a short form of the Berlin Intelligence Structure test. Two aspects of executive attention (sustained and selective attention) and speed of decision making were measured by a continuous performance test, a flanker task, and a Hick task, respectively. Traditional structural equation modeling, representing the positive manifold by a *g* factor, as well as network analyses, investigating the differential effects of the two aspects of executive attention and speed of decision making on the specific correlations of the positive manifold, suggested that selective attention, sustained attention, and speed of decision making explained the common but not the unique portions of the positive manifold. Thus, we failed to provide evidence for POT’s assumption that the positive manifold is the result of overlapping domain-general processes. This does not mean that domain-general processes other than those investigated here will not be able to show the pattern of results predicted by POT.

## 1. Introduction

### 1.1. Theoretical Background

One of the best-established and most replicated findings in psychology is the pattern of positive correlations among various tests of psychometric intelligence, even if these tests measure quite different mental abilities (van der Maas et al. 2006). This so-called positive manifold was first described by Spearman (1904). To account for this phenomenon, he proceeded from the assumption of a single underlying fundamental function, referred to as the *g* (or general) factor of intelligence or psychometric *g*. Later research corroborated the positive manifold but introduced additional group factors of intelligence to describe the positive manifold more adequately (Deary 2012). Group factor models describe intelligence as a hierarchical construct with specific tests or abilities at the lower level of the hierarchy, which can be grouped to more general factors at a higher level (e.g., fluid intelligence, crystallized intelligence, broad visual or auditory abilities). As these group factors are still correlated with each other, a *g* factor builds the apex of the hierarchy (Carroll 1993; Johnson and Bouchard 2005; McGrew 2009; Süß and Beauducel 2015).

Although most researchers agree on the possibility of describing the positive manifold (at least indirectly via group factors) by means of a *g* factor, the very meaning of this factor is still the subject of a lively debate. While originally Spearman (1904, 1927) assumed the *g* factor reflected mental energy, Jensen (1982) pointed to mental speed and Kyllonen (1996) to working memory capacity as possible sources underlying *g*. To date, however, no single function or process has been identified that fully accounts for the positive ma-nifold or correlates perfectly with *g*.

As early as 1916, Thomson demonstrated that a general factor can be extracted from a positive manifold even when, actually, no such unitary factor underlies the positive correlations among different tests (Thomson 1916). Thomson’s idea that the positive manifold can also be explained without the assumption of a *g* factor has been advanced and refined by further theoretical frameworks (Bartholomew et al. 2009; Detterman 2000; van der Maas et al. 2006). In a more recent approach, Kovacs and Conway (2016, 2019) proposed the *Process Overlap Theory* (POT). According to POT, all psychometric tests require a large number of different cognitive processes. Some processes are required primarily by tests of the same domain-specific ability (e.g., mental rotation for tests of spatial ability). These domain-specific processes *cause* the observation of group factors representing individual differences in these domain-specific abilities. Other processes are domain-general rather than domain-specific, i.e., they are required by tests from different domains, such as the verbal or the spatial domains. Kovacs and Conway (2016, 2019) suggested that processes of executive attention (also referred to as attention control or executive functions) are domain-general because these processes serve the activation or maintenance of goal-relevant information and the suppression of goal-irrelevant information during test completion—irrespective of whether information is processed in the verbal, spatial, or any other specific domain.

It is important to note that a given domain-general process is not necessarily required by all psychometric tests of a test battery. As long as each psychometric test shares a domain-general process with a test from another domain, this should lead to a positive ma-nifold—even if not a single domain-general process is required by *all* tests. This constellation should be sufficient to derive a *g* factor mathematically from the positive manifold (Detterman 2000). Obviously, this factor cannot be interpreted as a single *reflective factor* causally influencing performance on all tests. Rather, and this is a decisive point of POT, if psychometric *g* is the result of overlapping processes, it should be interpreted as a *formative factor*, which is a “summary statistic” (Kovacs and Conway 2019, p. 267) without psychological meaning.

POT implies several additional assumptions. Two major assumptions refer to the notion of a unique variance of *g* unrelated to overlapping processes and the non-additivity of the domain-specific and domain-general processes. Both these assumptions dissociate POT from similar previous theories, and they explain important phenomena in the realm of intelligence research, such as factor differentiation, or that more complex tasks show higher *g* loadings than less complex tasks (Kovacs and Conway 2019).

For the present study, Kovacs and Conway’s (2016) assumption of overlapping domain-general processes is of particular importance. This assumption means that each positive correlation (or at least most of them) in the positive manifold is *formatively caused* by other more or less independent domain-general processes or, in other words, by *different* aspects of executive attention. It is important not to confuse this assumption with the assumption that a single and general latent variable underlying different aspects of executive attention is related to psychometric *g* (cf. Kovacs and Conway 2016). In this latter case, the same basic function underlying psychometric *g* might also cause a positive manifold for different aspects of executive attention. Hence, the correlation between psychometric *g* derived from psychometric tests and general executive attention might then inform the amount of *g*-related variance shared by the psychometric tests and cognitive tasks. POT explicitly contradicts such a *g* theory-based explanation. This contradiction, however, also allows basic assumptions of POT to be put to a critical test. Based on these considerations, the aim of the present study was to empirically investigate whether different domain-general processes are either uniquely or commonly related to psychometric *g* and the correlations of the positive manifold.

### 1.2. The Present Study

In a previous study (Pahud et al. 2018), we assessed the functional relationship between aspects of psychometric intelligence and two kinds of executive attention—namely, sustained attention and selective attention. Both can be regarded as domain-general since both kinds of attention are needed to successfully complete cognitive tasks and psychometric intelligence tests—regardless of the cognitive domain. For the assessment of sustained attention, we used an adaptation of the continuous performance test (CPT; Halperin et al. 1991) with three conditions of increasing demands on sustained attention. Analogously, the demands on selective attention were experimentally increased across three conditions of an adapted Eriksen flanker task (Scheres et al. 2003). Beside these two experimental tasks, tapping two distinct aspects of executive attention, we also used a Hick task (Neubauer et al. 1992; Rammsayer and Brandler 2007) with three levels of task complexity (0-, 1-, and 2-bit condition). As indicated by increasing RTs from the 0- to the 2-bit condition, task complexity increases across the conditions. Within the conceptual framework of POT, complexity “refers to the extent to which a test taps executive/attentional processes” (Kovacs and Conway 2016, p. 164). Therefore, within the scope of POT, the increase in RT across the three Hick conditions as a function of increasing task complexity might be interpreted as an indicator of the demands on executive attention. On the other hand, the increasing RTs across the Hick conditions are a well-known function of the number of decisions to be made or the bits of information to be processed (Proctor and Schneider 2018). Thus, the Hick task is a measure of speed of choice or simple decision making as a function of increasing complexity levels (Jensen 1982), but it is not a genuine executive attention task. Proceeding from this view, the Hick task might be especially interesting to investigate whether aspects of executive attention relate differently to the correlations of the positive manifold compared to increasingly complex measures of speed of information processing (here speed of decision making)—broadly irrespective of executive attention.

Pahud et al. (2018) analyzed RTs in all three tasks by means of fixed-links modeling (Schweizer 2008). With this type of confirmatory factor analysis, two latent variables were extracted from each experimental task. One latent variable depicted variance in RT that did not vary as a function of the experimental manipulation and, therefore, was related to more general aspects of speed of information processing such as speed of sensory encoding or speed of motor execution. The other latent variable represented variance in RT, which systematically increased from the least to the most demanding condition. Thus, these latent variables reflected the increasing demands on sustained attention in the CPT, on selective attention in the flanker task, and speed of decision making in the Hick task.

In the study by Pahud et al. (2018) psychometric intelligence was assessed with a battery of 18 intelligence subtests of the Berlin Intelligence Structure (BIS) test (Jäger et al. 1997). This battery included six measures of processing capacity (broadly similar to reasoning), clerical speed, and memory, respectively, but abandoned the creativity measures, which represented the fourth operation in Jäger’s (1984) BIS model. A *g* factor of psychometric intelligence was extracted from indices of capacity, speed, and memory. In the present study, we expand these investigations by analyzing not only the *g* factor but also the positive manifold, i.e., the specific correlations among different aspects of intelligence in order to put POT to the test. A re-analysis of Pahud et al.’s data is of particular interest for this purpose since it contains the positive manifold of the psychometric tests as well as two different aspects of executive attention and a measure of speed of decision making. Thus, this data facilitates an investigation into whether different aspects of executive attention are differentially related to *g* and, most importantly, whether they contribute to the correlations among psychometric tests in the positive manifold to different degrees. More specifically, we investigated the following research questions:

1. Do different aspects of executive attention and speed of decision making uniquely or commonly explain variance in psychometric *g*? The approach to answering this question is in line with the traditional research on the *g* factor because *g* is more or less premised as an entity underlying the positive manifold.
2. In order to investigate the positive manifold more differentially, we employed network analyses to answer the question of how the correlations in the positive manifold change when considered concurrently with different aspects of executive attention and speed of decision making. These analyses focused on correlations in the positive manifold, from which the influence of executive attention was partialled out.

## 2. Materials and Methods

### 2.1. Participants

Pahud et al.’s (2018) sample consisted of 110 male and 118 female volunteers with an age range from 18 to 30 years. One hundred and four participants had no academic background. All participants reported normal or corrected-to-normal vision. The study was approved by the local ethics committee (2012-9-189242) and all participants gave their written informed consent prior to the testing.

### 2.2. Measurement of Psychometric Intelligence

A modified version of the short form of the Berlin Intelligence Structure (BIS) test was used to measure psychometric intelligence (Jäger et al. 1997). This test consisted of 18 subtests that are described in more detail in Pahud et al. (2018). BIS-Capacity, BIS-Speed, and BIS-Memory, representing individual performance in relation to reasoning, clerical speed, and memory (Oberauer et al. 2008), respectively, were assessed with six subtests each. Each set of the six subtests consisted of two verbal, two numerical, and two figural tests. For the purpose of the present study, each subtest was *z* standardized. Then, the standardized test scores of the six subtests per set were averaged to yield BIS-Capacity, BIS-Speed, and BIS-Memory scores. In an unpublished study (Wicki 2014), the retest reliability coefficients for the BIS-Capacity, BIS-Speed, and BIS-Memory scores in this adapted short form were *r*_tt_ = .79, *r*_tt_ = .85, and *r*_tt_ = .86, respectively (N = 122; test retest interval = 1 month). The retest reliability of the *g* factor derived from this modified short-form of the BIS test was *r*_tt_ = .79.

### 2.3. Experimental Tasks

All tasks were fully computer controlled and programmed in Eprime 2.0. Stimuli were presented on a computer monitor and responses were registered via an external Cedrus© key pad with a temporal resolution of ±1ms. All tasks were preceded by verbal and written instructions and practice trials. A very detailed description of the experimental tasks is provided in Pahud et al. (2018).

### 2.4. Flanker Task

To assess selective attention, a version of Eriksen’s flanker task was adapted from Scheres et al. (2003) with three task conditions. On each trial of the first and the second condition, an arrow was presented on the monitor screen. This arrow could point to the right- or to the left-hand side. Participants had to respond to the arrow by pressing a designated response key as fast as possible. In the 32 trials of the first condition, the direction of the arrow was not to be attended to. In the 32 trials of the second condition, participants were required to respond with the forefingers of the right and the left hand when the arrow pointed to the right or the left side, respectively. As in the second condition, participants had to respond to the direction of the arrow in the third condition. Here, however, the arrow was flankered by additional arrows, which pointed congruently to the same direction or incongruently to the opposite direction. The third condition consisted of 32 trials with congruent flankers and 32 trials with incongruent flankers. In all conditions, the next trial started 500 ms after the completion of the response to the preceding trial. As a measure of performance, individual mean RTs of correct responses were obtained for each condition. With its demands to selectively attend to relevant information and to inhibit irrelevant information, the flanker task is a well-established measure in the field of selective attention (Scheres et al. 2003) and attention control (Draheim et al. 2021; Shipstead et al. 2014).

### 2.5. Continuous Performance Test

The present version of the CPT was adapted from Halperin et al. (1991). In all three conditions of the CPT, stimuli were presented on the monitor screen for 200 ms, followed by a 1000-ms interval with a black screen, before the next trial started. In the 32 trials of the first condition, only the letter “X” was presented and participants were instructed to respond as fast as possible to its onset. The second condition consisted of 120 trials with different letters. In 96 trials, distractor letters (K, D, W, R, S, M, G, and A) did not require a response. In the remaining 24 trials, an “X” was presented and participants had to respond as fast as possible to this target letter. The same stimuli were used in the third condition, which consisted of 240 trials with 196 distractor letters. The letter “X” was presented in normal font (24 trials) or italic font (24 trials). Participants were told that they should only respond to the italic “*X*” as fast as possible. As dependent variables, individual mean RTs of correct answers in the three CPT conditions were determined. The CPT is one of the best-established measures of sustained attention and vigilance, for which reliability and validity have been demonstrated repeatedly (Raz et al. 2014; Riccio et al. 2002).

### 2.6. Hick Task

A version of the Hick task, adapted from Neubauer et al. (1992), was used which measures the speed of making simple decisions or choices. The task consisted of a 0-bit, a 1-bit, and a 2-bit condition. In the 0-bit condition, a trial started with the presentation of a rectangle in the center of the computer monitor. After a foreperiod, which varied randomly between 1000 and 2000 ms, a “+” sign appeared in the rectangle and participants’ task was to press a designated key as fast as possible after the onset of the “+” sign. In the 1-bit condition, two rectangles were presented next to each other on the monitor and the “+” sign appeared after the fore period in the right or the left rectangle. Participants pressed a right or a left key (with the right or the left forefinger) corresponding to the rectangle, in which the “+” sign was presented. In the 2-bit condition, four rectangles were presented (in two lines and two columns) and participants responded with the forefingers and middle fingers of their right and left hand corresponding to the rectangle, in which the “+” sign was presented. For each condition, the mean RT of the correct answers was determined.

### 2.7. Statistical Analyses

The analyses will be outlined during the course of the results section for reasons of comprehensibility. All analyses were carried out with RStudio (psych package for descriptive statistics and correlational analyses; lavaan package for confirmatory factor analyses and structural equation modeling; SEM; qgraph and psychonetrics for network analyses). The estimator in SEM was maximum likelihood with Satorra-Bentler correction. For the evaluation of model fit, the following fit indices were used (evaluation criteria given in parenthesis according to Schreiber et al. 2006): χ^2^ (ratio of χ^2^ to df < 3), robust comparative fit index (CFI_rob_ > .950), root mean square error of approximation (RMSEA < .080), and standardized root mean square residual (SRMR ≤ .08). The data set can be requested under www.osf.io/jgxsr (doi:10.17605/OSF.IO/JGXSR, accessed on 23 June 2021).

## 3. Results

**Figure 1:**
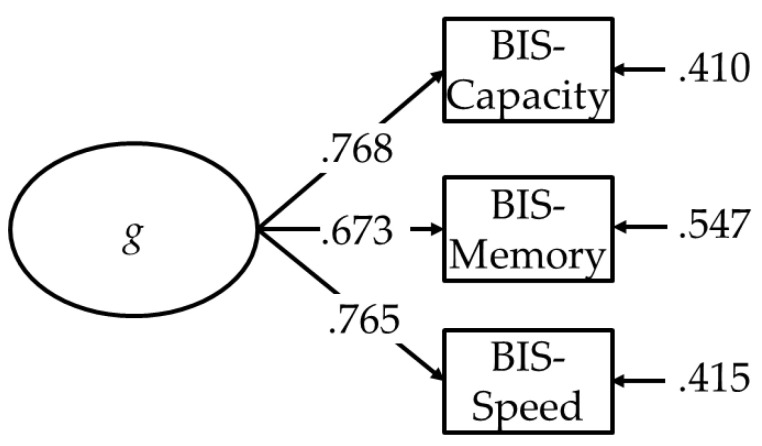
The *g* factor extracted from the average scores of BIS-Capacity, BIS-Memory, and BIS-Speed tests.

The three aspects of psychometric intelligence, BIS-Capacity, BIS-Speed, and BIS-Memory correlated significantly with each other. The correlation coefficients are presented in Table 1. Figure 1 shows the *g-*factor model with *g* extracted from the three aspects of psychometric intelligence. As a measure of reliability, McDonald’s omega coefficient was Ω = .782 for the *g* factor.

**Figure 2:**
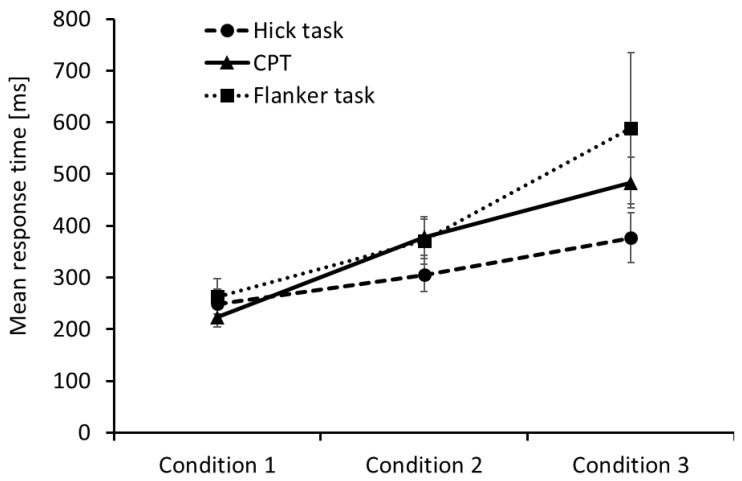
Mean response times in the three conditions of the flanker task, the continuous performance test (CPT), and the Hick task, respectively. Error bars represent the corresponding standard deviations.

Mean RTs and the corresponding standard deviations in the three conditions of the flanker task, the CPT, and the Hick task, respectively, are presented in Figure 2. As reported in Pahud et al. (2018), the RT differences within each task were statistically significant, indicating that the experimental manipulation led to increasing demands on selective attention in the flanker task, to increasing demands on sustained attention in the CPT, and to increasing response latencies as a function of the number of decisions in the Hick task.

**Figure 3:**
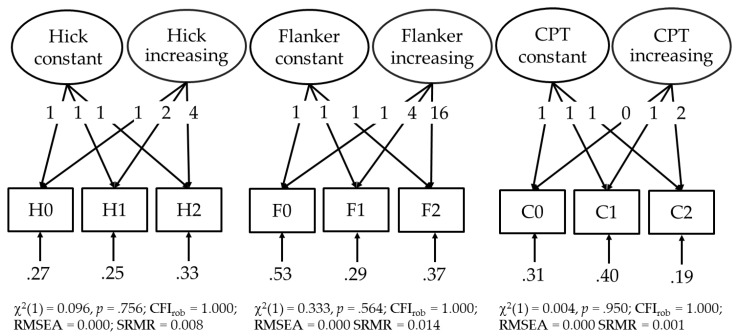
Fixed-links models for the Hick task, the flanker task, and the continuous performance test (CPT). Unstandardized factor loadings are presented as well as model fit by the χ^2^ test, the robust comparative fit index (CFI_rob_), the root mean squared error of approximation (RMSEA), and the standardized root mean square residual (SRMR).

For each task, the RTs in the three task conditions were analyzed by means of fixed-links modeling. With this kind of confirmatory factor analysis, the effect of the experimental manipulation of the task demands can be dissociated from variability in the data that did not vary with the manipulation (Schweizer 2009). Thus, for each task, two latent variables were extracted. For one latent variable, factor loadings were fixed to “1” to depict individual differences in the RT that did not vary as a function of the task demands and, hence, individual differences in the task-unspecific aspects of speed of information processing (e.g., sensory encoding or motor execution) were captured. The second latent variable had factor loadings, which increased monotonically from the easiest to the most demanding task condition. This increasing variability in the RT data reflects individual differences in selective attention, sustained attention, and in speed of decision making, respectively. The increasing trajectories of the factor loadings of the latent variables representing selective attention, sustained attention, and speed of decision making are presented in Figure 3. The RT data of all three tasks could be adequately described by this statistical procedure as indicated by the model fit indices reported in Figure 3. Since all factor loadings were fixed in these models, the variances of the latent variables were estimated. The variances of the latent variables with increasing factor loadings (*p* < .001) as well as those with constant factor loadings (*p* < .05) were statistically significant. Furthermore, reliability analyses resulted in estimations of the McDonald’s Ω of .816, .753, and .770 for the increasing latent variables in the CPT, the Hick, and the Flanker task, respectively, and Ω = .760, .808, and .401 for the constant latent variables.

**Figure 4:**
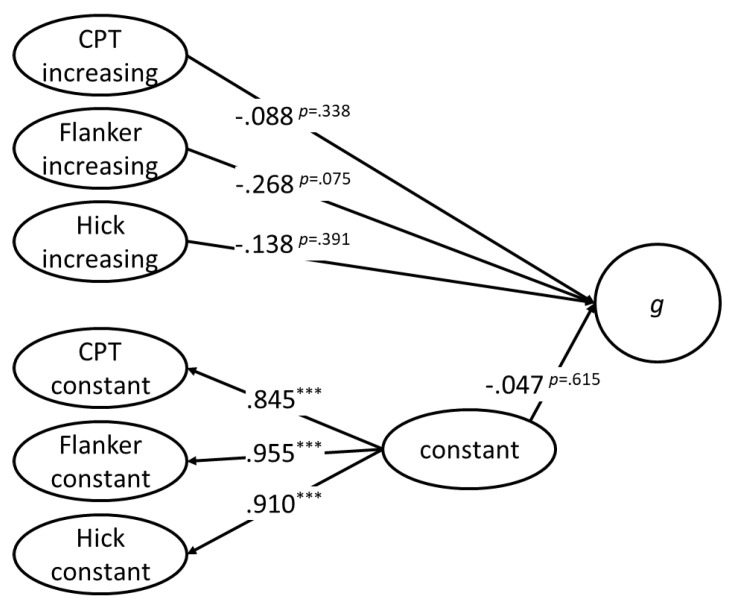
Latent regression analysis of the relationship between the *g* factor and the latent variables extracted from the flanker task, the continuous performance test (CPT), and the Hick task. Model fit: χ^2^_SB_(44) = 102.254; *p* < .001; CFI_rob_ = .947; RMSEA = .076; SRMR = .056. *** *p* < .001.

After having established measurement models for psychometric *g*, selective and sustained attention as well as speed of decision making, the following analyses aimed to answer the first research question of whether the two aspects of executive attention and speed of decision making uniquely or commonly explain variance in psychometric *g*. All latent variables with increasing factor loadings correlated with the *g* factor of psychometric intelligence (see Table 2). A different picture emerged for the latent variables with constant factor loadings, where only the latent variable extracted from the flanker task correlated significantly with *g*. In the next step, we investigated the unique variance in *g* explained by these latent variables. Due to very high correlations between the latent variables with constant factor loadings and to avoid collinearity-related issues, these variables were reduced to one latent variable before *g* was regressed on this latent variable together with the three latent variables with increasing factor loadings (see Figure 4). The model described the data well according to all fit indices except the CFI (.947), which fell below the criterion value of .950. Neither the latent variables reflecting selective and sustained attention, respectively, nor those reflecting speed of decision making or more basic speed of information processing explained unique portions of variance in *g* above and beyond the variance explained by the other latent variables. Given the results reported in Table 2, this pattern of results indicated that the portion of the variance of *g* explained by any of the latent variables from our three tasks was the same portion explained by the other latent variables.

**Figure 5:**
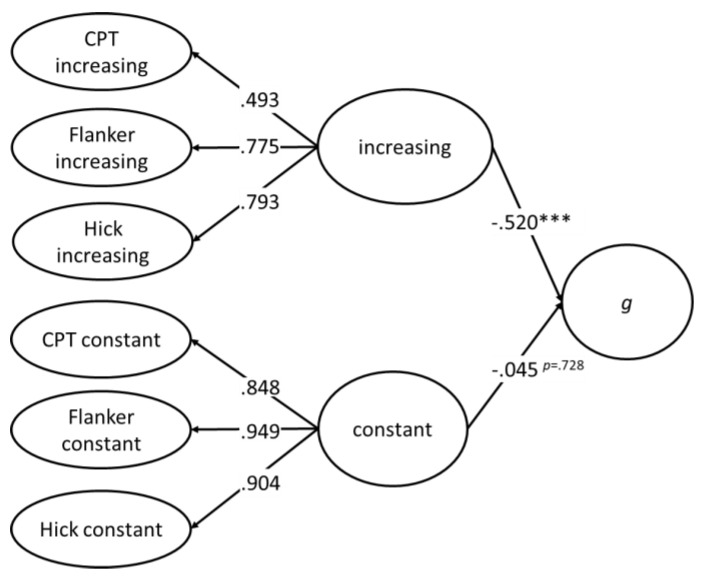
Latent regression analysis of the relationship between the *g* factor and two second-order latent variables extracted from the flanker task, the continuous performance test (CPT), and the Hick task. Model fit: χ^2^_SB_(48) = 104.243; *p* < .001; CFI_rob_ = .949; RMSEA = .072; SRMR = .056. *** *p* < .001.

In other words, the portions of the variance in *g* explained by selective attention, sustained attention, and speed of decision making, respectively, overlapped. To further elaborate on this idea, a second-order latent variable was derived from the three latent variables with increasing factor loadings and the three latent variables with constant factor loadings, respectively. Then, these two second-order latent variables were combined with *g* in a structural equation model (see Figure 5). The model data fit was acceptable except for the CFI (.949), which just failed to reach the criterion of .950. While the second-order latent variable representing the increasing complexity across the three tasks was substantially related to *g* (β = −.52, *p* < .001), the second-order latent variable representing individual differences in the basic aspects of speed of information processing was not associated with *g* (β = .045, *p* = .726).

Proceeding from the model depicted in Figure 5, we tested whether one of the first-order latent variables with increasing factor loadings could explain portions of variance in *g* above and beyond the variance explained by the second-order latent variable. When simultaneously considering all three additional regressions, the process of estimation did not converge so that three separate models were computed. In these models, the regression coefficients were β = −.140, *p* = .444, and β = .001, *p* = .995 for the flanker task and the continuous performance test, respectively. No model explained the data better than the more parsimonious model without these additional regressions. For the Hick task, the model did not converge.

In sum, these analyses, including the *g* factor of psychometric intelligence as a latent variable representing the positive manifold, did not provide evidence for the notion that selective and sustained attention as two different aspects of executive attention and speed of decision making differentially predicted *g* as a representation of the positive manifold. Rather, the different aspects of executive attention appeared to have a substantial amount of variance in common, which they also shared with speed of decision making and, eventually, with *g*.

Previous analyses proceeded from the assumption of the *g* factor as an entity underlying the positive manifold. POT, however, is less concerned with accounting for the representation of all correlations in the positive manifold. Instead, it assumes that various aspects of executive attention uniquely explain these correlations. Therefore, the following analyses focused on the correlations among BIS-Capacity, BIS-Speed, and BIS-Memory depending on variables reflecting aspects of executive attention, speed of decision making, and basic speed of information processing. For this purpose, we applied a network analysis approach (Epskamp et al. 2012) in which the variables under investigation are depicted as nodes and the strength of the relationships among these variables are represented by the distance between the nodes and the thickness of the edges connecting the variables. For the present purpose, the possibility of investigating partial correlations was of particular interest. As each partial correlation in the network analysis is controlled for the influence of all other variables in the network, changes in the positive manifold due to integrating one or more aspects of executive attention can be easily depicted.

**Figure 6:**
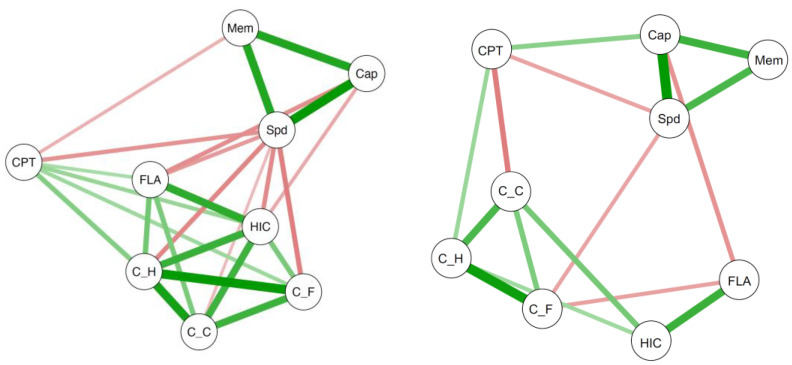
Network analysis based on Pearson correlations (left panel) and partial correlations (right panel) between three aspects of psychometric intelligence (BIS-Capacity, BIS-Speed, and BIS-Memory), sustained attention, selective attention, and speed of decision making, as well as three variables representing basic speed in the three experimental tasks. Green and red edges indicate positive and negative relationships, respectively. Only the (partial) correlations that reached statistical significance (*p* < .05) are presented. Abbreviations: Cap = BIS-Capacity; Mem = BIS-Memory; Spd = BIS-Speed; CPT = factor scores of the latent variable with increasing factor loadings from the continuous performance test (sustained attention); FLA = factor scores of the latent variable with increasing factor loadings from the flanker task (selective attention); HIC = factor scores of the latent variable with increasing factor loadings from the Hick task (speed of decision making); C_C, C_F and C_H = factor scores of the latent variables with constant factor loadings from the CPT (C_C), the flanker task (C_F), and the Hick task (C_H).

From the above described fixed-links models for the experimental tasks, we extracted factor scores for “sustained attention”, “selective attention”, and “speed of decision making”, as well as for the three constant latent variables representing basic speed. Together with BIS-Capacity, BIS-Speed, and BIS-Memory, these six factor scores were submitted to a first network analysis based on Pearson correlations. The result of this analysis is shown in the left panel of Figure 6, with green and red edges representing significant positive and negative correlations, respectively. The three BIS variables clustered together and, similarly, the three variables of basic speed were also close together. In contrast to the basic speed variables, the variables representing different aspects of executive attention and speed of decision making were less unambiguously clustered, but closer to the intelligence cluster than the basic-speed variables. Interestingly, sustained attention was somewhat separated despite its negative associations with BIS-Memory and BIS-Speed. Moreover, while BIS-Memory was only related to sustained attention, both BIS-Capacity and BIS-Speed were related to selective attention and speed of decision making. Finally, all variables representing basic speed were significantly and negatively related to BIS-Speed but not to BIS-Capacity or to BIS-Memory.

To investigate the partial correlations, we used a step-wise procedure. In the first step, the variance shared by all three aspects of intelligence was partialled out so that the partial correlations reflected the variance, which was shared pairwise by BIS-Memory and BIS-Speed, BIS-Capacity and BIS-Speed, and BIS-Memory and BIS-Capacity, respectively. As can be seen from the first line of partial correlations in Table 1, the partial correlations between BIS-Capacity, BIS-Speed, and BIS-Memory were substantially lower than the corresponding Pearson correlations (see the first numbered line in Table 1 with the 95% confidence intervals of these partial correlations reported in parentheses). In the next three steps, either selective attention, sustained attention, or speed of decision making were added to the model, respectively. The numbered lines 2 to 4 of Table 1 show the resulting partial correlations, which hardly changed compared to the model, without controlling for the influence of executive attention variables and speed of decision making, given that none of these partial correlations were lower than the lower bounds of the confidence intervals of the respective partial correlation in the first step.

Then, the combinations of two aspects of executive attention/speed of decision making were included in the analyses (see numbered lines 5–7 of the partial correlations in Table 1). These partial correlations were not substantially lower compared to the partial correlations when only selective attention, sustained attention, or speed of decision making were considered solely. A similar result was obtained when selective and sustained attention as well as speed of decision making were controlled for simultaneously (see numbered line 8 of the partial correlations in Table 1).

When only the factor scores of the three latent variables with constant factor loadings were controlled for, the partial correlations were again very similar to the previous partial correlations (see numbered line 9 of the partial correlations in Table 1). Eventually, a virtually identical pattern of results was obtained for the final model, in which all the variables were controlled for (see numbered line 10 of the partial correlations in Table 1). Again, the partial correlations in this final model were not lower than the lower bounds of the confidence intervals of the partial correlations in the first model. Thus, regardless of the type of controlled processes and, most surprisingly, irrespective of the number of controlled processes, the positive manifold always changed in a very similar way.

The final network analysis based on the partial correlations and including all the variables is depicted in the right panel of Figure 6. The three aspects of intelligence as well as the three aspects of basic speed still clustered together. Selective attention and speed of decision making in the Hick task were also still close to each other, while both lost their relationship to sustained attention. Furthermore, selective attention was still related to BIS-Capacity, but no longer to BIS-Speed. As in the correlation-based network analysis, selective attention was unrelated to BIS-Memory and sustained attention was related to BIS-Speed—even when all other influences were controlled for in the partial-correlation-based network analysis. Finally, speed of decision making as well as all three variables of basic speed were unrelated to aspects of psychometric intelligence, when the variance shared with all other variables was controlled for.

## 4. Discussion

The present study examined the relationship between two different aspects of executive attention (selective attention and sustained attention), speed of decision making and the positive manifold of correlations between different aspects of psychometric intelligence (BIS-Capacity, BIS-Speed, and BIS-Memory). For this purpose, two different approaches were applied. With the first approach, the positive manifold was represented by a *g* factor to probe whether different aspects of executive attention explain unique or common portions of variance in *g*. Although both aspects of executive attention as well as speed of decision making were significantly related to *g*, we found no evidence that one of them explained portions of variance in *g* above and beyond the variance explained by the others. The second approach used network analyses with partial correlations to investigate the influence of the two aspects of executive attention and speed of decision making on the single correlations of the positive manifold. Partial correlations were computed between BIS-Capacity and BIS-Memory, BIS-Capacity and BIS-Speed, as well as BIS-Memory and BIS-Speed with the variance common to all three aspects of intelligence partialled out. Then, the influence of sustained and selective attention as well as speed of decision making on these partial correlations was investigated. Neither sustained or selective attention nor speed of decision making substantially affected these relationships. Hence, both approaches failed to provide evidence for POT’s assumption that the positive manifold is the result of overlapping domain-general processes.

Within the conceptual framework of POT, Kovacs and Conway (2016) assumed that the *g* factor does not represent an entity but is the result of different aspects of executive attention causing (more or less pairwise) correlations between different aspects of intelligence such as BIS-Capacity, BIS-Speed, and BIS-Memory. As has been shown by Detterman (2000), the positive entries of a correlation matrix—even if not caused by the same single mechanism or process—represent a sufficient condition for the extraction of a *g* factor. Therefore, according to POT, the long-lasting search for Spearman’s basic function underlying the *g* factor has not been successful because there is no such basic function. Instead, different domain-general processes contribute differentially to the correlations of the positive manifold. Executive functions (or aspects of executive attention) are especially emphasized by POT because they refer to the activation or the maintenance of goal-relevant information and to the suppression of goal-irrelevant information. These aspects of executive attention are required by various tasks, even when these tasks belong to different group factors (e.g., spatial and verbal abilities). For this reason, they are considered domain-general and should cause correlations between otherwise distinct areas of information processing. POT’s core assumption of overlapping processes causing the *g* factor in a formative (rather than a reflective) way is enthralling and plausible but certainly needs empirical validation.

In the present study, we started the investigation with a traditional approach to the positive manifold by extracting a *g* factor from BIS-Capacity, BIS-Speed, and BIS-Memory. In all three experimental tasks, the means and variances of RTs increased from the simple to the most complex task condition, pointing to increasing demands on executive attention in the CPT and the flanker task, and an increasing number of decisions in the Hick task. By means of fixed-links modeling, selective attention and sustained attention were represented by latent variables, which described the increasing variance across the three conditions of the flanker task and the CPT, respectively. In the Hick task, the latent variable with increasing factor loadings from the 0-bit to the 2-bit condition was interpreted to reflect speed of decision making. The fixed-links modeling approach also allowed for the control of the more basic aspects of speed of information processing, which were functionally independent of the experimental manipulation and reflected processes such as speed of stimulus encoding and motor execution.

Combining the *g* factor model of intelligence with the measurement models for the CPT, flanker task, and Hick task revealed that sustained attention, selective attention, as well as speed of decision making were indeed related to *g*. Of particular interest for the present purpose was the finding that neither sustained or selective attention nor speed of decision making explained portions of *g* variance independently from the other two aspects. Sustained and selective attention as well as speed of decision making shared a substantial amount of variance. Furthermore, this common variance facilitated the extraction of a higher-order latent variable, which shared about 25% of the variance in *g*. Neither sustained or selective attention nor speed of decision making explained further variance in *g* beyond the amount explained by the higher-order latent variable. Our finding of a substantial portion of shared variance between aspects of executive attention and intelligence is consistent with a large number of previous reports (Friedman and Miyake 2017; Friedman et al. 2008; Tsukahara et al. 2020). It should be noted though that other studies have failed to establish a higher-order latent variable (e.g., Friedman et al. 2006; Rey-Mermet et al. 2019).

The finding that the three latent variables representing different aspects of executive attention and speed of decision making shared a substantial portion of common variance as well as the fact that this common variance was shared with *g* contradicted the above-mentioned assumptions of POT. The representation of the positive manifold by the *g* factor, however, might have concealed the fact that single correlations in the positive manifold are differentially related to aspects of executive attention (and speed of decision making) as proposed by POT. To probe this idea, we employed a network analysis approach based on partial correlations. It was of particular interest in these analyses whether single correlations between aspects of psychometric intelligence would differentially decrease when controlled for one or more aspects of executive attention. The outset was quite promising for these analyses because selective attention correlated significantly with all three aspects of psychometric intelligence, but sustained attention only correlated with BIS-Memory and BIS-Speed, and speed of decision making in the Hick task only correlated with BIS-Capacity and BIS-Speed. At first sight, this pattern of results might be considered as supporting evidence for POT. The partial correlations, however, revealed that none of the correlations between pairs of aspects of intelligence were substantially decreased when controlled for selective or sustained attention or speed of decision making. Thus, similar to the outcome obtained in the investigation of *g*, the results of the network analyses did not support the assumptions of POT. Rather, our results indicate that different aspects of executive attention have something in common, which is also shared by speed of decision making. This communality appeared to be related to the common variance of all aspects of psychometric intelligence which means to *g*.

With the fixed-links modeling approach, we aimed to measure aspects of executive attention and speed of decision making and, concurrently, to control for more basic aspects unrelated to the experimental manipulation of task demands such as speed of stimulus encoding and motor execution. Nevertheless, the focus on RT measures in the present study might be considered a limitation as the same method of measurement can easily lead to a bias, such as speed-accuracy trade-offs, which can only be controlled for when different methods of measurement are used. From this point of view, a more heterogeneous battery of executive attention tasks is recommended for future studies. Such a battery could tap, for example, the most common taxonomy of executive attention that is based on the work by Friedman and Miyake (Friedman et al. 2006; Friedman et al. 2008) with the three basic functions referred to as “working-memory updating”, “pre-potent response inhibition”, and “task-set shifting” (Friedman and Miyake 2017). However, extensions of this framework by relational integration (Himi et al. 2021) or more fine-grained investigations of different aspects of inhibition (Rey-Mermet et al. 2018) might also be promising to examine whether other aspects of executive attention—primarily when based on error rates rather than RT measures—differentially account for correlations in the positive manifold.

The high correlation between selective and sustained attention as two aspects of executive attention might be surprising given that previous research pointed to distinct attentional and neural networks underlying selective and sustained attention (e.g., Fan et al. 2002; Raz and Buhle 2006). The correlational approach in the present study, however, is not well suited to decide whether two or more processes are distinct. If a biological characteristic of the brain such as white matter integrity leads to individual differences in general brain functioning (Penke et al. 2012), individuals with better function in one network will probably also show better function in other networks. Garlick (2002) put forward a similar (and more elaborated) idea to explain the positive manifold of psychometric intelligence by means of neural plasticity. Proceeding from the assumption of such a brain characteristic (whether white-matter integrity, neural plasticity, or any other), which affects most or all brain regions, individual differences in most or all kinds of information processing should be affected by this characteristic. More precisely, if the completion of a task needs a certain number of single processes and each process is affected by this brain characteristic, then it is reasonable to assume that the performance in this task will be more affected than the performance in another task that requires fewer processes. As a result, performances in tasks requiring a higher number of processes should be more strongly associated with each other rather than performances in tasks requiring a lower number of processes. Such a pattern became evident in the present data and is depicted in Figure 6, since the strongest correlations were obtained among the three aspects of psychometric intelligence with each aspect comprising a high number of different processes. Correlations between psychometric intelligence and aspects of executive attention as well as speed of decision making were lower—maybe due to the lower number of processes required by the experimental tasks compared to the psychometric intelligence tests. The correlations among sustained and selective attention as well as speed of decision making, in turn, were in a similar range as those between these variables and aspects of intelligence. This suggests again that the number of required processes might be the limiting factor. Finally, the weakest correlations were found between aspects of psychometric intelligence and the latent variables representing basic aspects of speed of information processing and, therefore, representing the lowest number of processes.

Our executive attention and speed of decision making latent variables might therefore just describe the increasing number of processes required across the three conditions of each task, representing the core of task complexity. From this point of view, our results are in line with the complexity hypothesis (e.g., Stankov 2000; Vernon and Weese 1993), which holds that an increase in the complexity of an RT task leads to a stronger correlational relationship between RT and psychometric intelligence—regardless of the specific operations required by the RT task. This would also explain why increasing the number of decisions in the Hick task led to a similar effect for the RT–intelligence relationship as the increase in the demands on executive attention in the CPT and the flanker task. It should be noted, however, that the interpretation of task complexity on the basis of the number of the required processes differs from the one proposed by POT. According to POT, complexity “refers to the extent to which a test taps executive/attentional processes” (Kovacs and Conway 2016, p. 164) and aspects of executive attention are conceptualized as top-down processes enabling goal-oriented behavior. Oberauer (2016) casts doubt on the assumption of processes, which are more general than others, since those processes are not considered in most models of cognitive control. Our results can be easily and quite parsimoniously explained by complexity as defined by the number of processes required for successfully performing a given task. A more systematic investigation of this idea, however, would require a more balanced selection of tasks with several tasks on different aspects of executive attention as well as different tasks in which—similar to the Hick task—the number of (the same) processes to be executed is manipulated to contrast their prediction of *g* or correlations in the positive manifold.

Finally, the fact that the aspects of executive attention, speed of decision making, and their interplay with aspects of psychometric intelligence did not support the assumptions made by POT does not necessarily mean that other aspects of executive attention will also fail to show a pattern of results as predicted by POT. Even if the present results were not consistent with POT, a rejection of POT would be premature. Therefore, our study should be considered as a paradigmatic proposal of how to examine major assumptions of POT rather than a (final) judgment of its validity.

## Figures and Tables

**Table 1:** Coefficients of Pearson and partial correlations between BIS-Capacity, BIS-Speed, and BIS-Memory.

	*r*_Memory-Speed_	*r*_Capacity-Speed_	*r*_Capacity-Memory_
Pearson correlation	.515	.588	.517
Partial correlations controlled for…			
1. the third aspect of intelligence, respectively.	.305 (.182–.418)	.438 (.326–.538)	.309 (.187–.423)
2. latent variable “selective attention”	.310	.408	.316
3. latent variable “sustained attention”	.266	.459	.322
4. latent variable “speed of decision making”	.318	.409	.315
5. latent variables “selective attention” and “sustained attention”	.271	.433	.333
6. latent variables “selective attention” and “speed of decision making”	.319	.400	.316
7. latent variables “sustained attention” and “speed of decision making”	.282	.430	.333
8. latent variables “selective attention”, “sustained attention”, and “speed of decision making”	.282	.423	.335
9. latent variables “basic speed” (constant factor loadings)	.325	.426	.303
10. all latent variables	.300	.421	.321

Note. All correlations were statistically significant (*p* < .001). Values in parentheses refer to the 95% confidence interval.

**Table 2:** Correlations between different aspects of intelligence (BIS-Capacity, BIS-Memory, BIS-Speed as well as the g factor extracted from these three aspects) and the latent variables derived from the flanker task, the continuous performance test (CPT) and the Hick task.

	Capacity	Memory	Speed	*g*
Flanker_increasing_ (selective attention)	−.330 ***	−.211 ***	−.259 ***	−.337 ***
CPT_increasing_ (sustained attention)	−.058	−.230 **	−.256 ***	−.245 **
Hick_increasing_ (speed of decision making)	−.287 **	−.153	−.296 **	−.288 **
Flanker_constant_	−.044	.045	−.283 *	−.206 *
CPT_constant_	.037	.157 *	−.104	−.091
Hick_constant_	−.014	.028	−.182 **	−.159

Note. “increasing” and “constant” refer to latent variables with increasing and constant factor loadings, respectively. * *p* < .05; ** *p* < .01; *** *p* < .001.

## Data Availability

Data are available at https://osf.io/jgxsr, accessed on 23 June 2021.
